# Natural Polymers-Based Materials: A Contribution to a Greener Future

**DOI:** 10.3390/molecules27010094

**Published:** 2021-12-24

**Authors:** Ana C. Q. Silva, Armando J. D. Silvestre, Carla Vilela, Carmen S. R. Freire

**Affiliations:** Department of Chemistry, CICECO–Aveiro Institute of Materials, University of Aveiro, 3810-193 Aveiro, Portugal; ana.cristina.silva@ua.pt (A.C.Q.S.); armsil@ua.pt (A.J.D.S.); cvilela@ua.pt (C.V.)

**Keywords:** natural polymers, polysaccharides, proteins, green chemistry, sustainability, composites and hybrid materials, films and membranes, patches, nanosystems, microneedles

## Abstract

Natural polymers have emerged as promising candidates for the sustainable development of materials in areas ranging from food packaging and biomedicine to energy storage and electronics. In tandem, there is a growing interest in the design of advanced materials devised from naturally abundant and renewable feedstocks, in alignment with the principles of Green Chemistry and the 2030 Agenda for Sustainable Development. This review aims to highlight some examples of the research efforts conducted at the Research Team BioPol4fun, Innovation in BioPolymer-based Functional Materials and Bioactive Compounds, from the Portuguese Associate Laboratory CICECO–Aveiro Institute of Materials at the University of Aveiro, regarding the exploitation of natural polymers (and derivatives thereof) for the development of distinct sustainable biobased materials. In particular, focus will be given to the use of polysaccharides (cellulose, chitosan, pullulan, hyaluronic acid, fucoidan, alginate, and agar) and proteins (lysozyme and gelatin) for the assembly of composites, coatings, films, membranes, patches, nanosystems, and microneedles using environmentally friendly strategies, and to address their main domains of application.

## 1. Introduction

Natural polymers, such as polysaccharides, proteins, and nucleic acids, are components of biological systems responsible for performing a wide range of essential functions [1]. For instance, certain natural polymers play a key role in the maintenance of the structural integrity of cells in plants and animals (e.g., cellulose [2] and chitin [3]), while others offer biological protection against surrounding environments (e.g., lysozyme [4]). The diversity in terms of provenance and composition provides these natural polymers with distinct physicochemical and biological properties that can be of interest in various fields [5,6]. In fact, natural polymers and their derivatives already find application in numerous sectors, e.g., in the manufacture of paper goods [7] and textiles [8], as additives in food products [9,10,11], in the formulation of nutraceuticals and functional foods [12], and in the biomedical field (e.g., in cosmetic treatments [13] and drug delivery [14,15]). Owing to the natural abundance, renewability, and intrinsically negative carbon footprint of polymers derived from renewable resources [16], their exploitation is favourable and can play a pivotal role in the development of advanced materials in the shape of films [17,18], membranes [19,20], coatings [21,22], hydrogels [23], and micro- and nanoparticle systems [24,25,26].

Over the last two decades, the movement towards greener and more sustainable practices has gained momentum, and much interest has been devoted to the exploitation of naturally abundant feedstocks that can be accessed without competing with natural food supplies [27,28,29], as a path to reducing the massive consumption of fossil resources, associated with reserve depletion and environmental concerns. The use of sustainable raw materials has become one of the fundamental goals of Green Chemistry and is described in the original set of twelve rules known as “The Twelve Principles of Green Chemistry” [30], which are set out in detail in a review by Anastas and Eghbali [31]. This mindset has been supported by the enforcement of environmental regulations, such as the Pollution Prevention Act of 1990 that is enforced by the US Environmental Protection Agency (EPA), the Rio Declaration on Environment and Development that was adopted by the United Nations (UN) Conference on Environment and Development only two years later [32], and the adoption of the UN objectives of the 2030 Agenda for Sustainable Development [33]. The role of academia in the implementation of more sustainable practices is evident in the upsurge of diverse literature reports dealing with the maximization of resources [34,35], the use of environmentally benign solvents [36,37,38], and the valorisation of renewable resources for the recovery of high-value low-molecular-weight compounds and macromolecular fractions [39,40] (to give a few examples).

Given the relevance of academic circles in the advocacy of Green Chemistry practices, the present appraisal aims to provide a brief overview of the research carried out by the BioPol4fun research group, viz. Innovation in BioPolymer-based Functional Materials and Bioactive Compounds, from the Portuguese associate laboratory CICECO–Aveiro Institute of Materials [41] at the University of Aveiro in Portugal. Among other research topics [42,43,44,45], this interdisciplinary group exercises research activities devoted to the use of renewable feedstocks for the extraction of high-value compounds from agroforest and industrial by-products [39,40,46], the valorisation of vegetable oils for the production of monomers and polymers [47,48,49,50,51], the design of biobased polyesters [52,53,54,55], and, in particular, the development of a panoply of natural polysaccharide- and protein-based materials [6,56,57,58,59,60,61,62].

**Figure 1:**
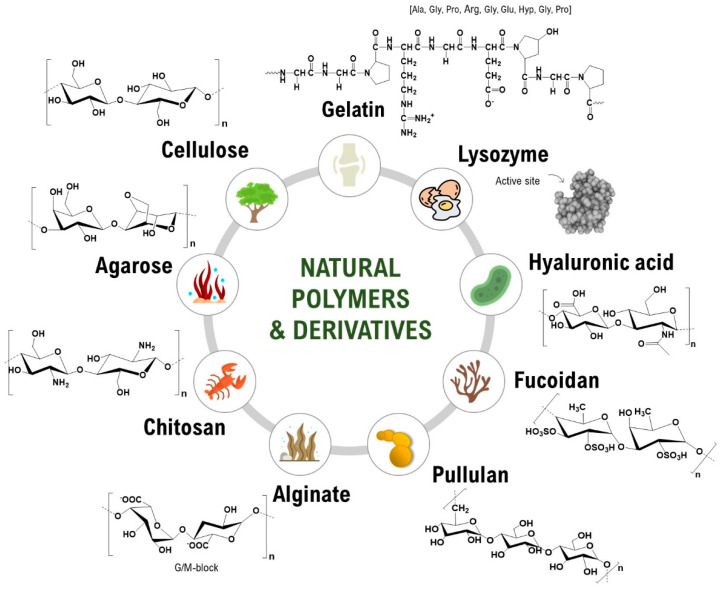
Examples of the natural polymers and derivatives used for materials design by the BioPol4fun research group.

Considering the scope of this review, we have chosen to focus on key publications of the last decade resulting from the journey of the BioPol4fun research group towards the development of innovative, sustainable, and biobased polymeric materials, in line with Principle 7 of Green Chemistry (“Use of renewable feedstocks”) and Goal 12 of the 2030 Agenda for Sustainable Development (“Ensure sustainable consumption and production patterns”). Some contributions in the development of different sustainable and environmentally friendly materials, viz. films, membranes, patches, coatings, nanosystems, and microneedles, derived from polysaccharides and proteins (Figure 1), will be highlighted and their main fields of application discussed. Finally, we conclude this review with an overview of the role of natural polymers in materials science and the significance of Green Chemistry in the sustainable design of biobased materials.

## 2. Natural Polymers-Based Materials at the BioPol4Fun Research Group

Engineering natural polymers-based materials derived from polysaccharides and proteins is the main objective of the work carried out by the BioPol4fun research group. In the last decade, we have been intensively exploiting cellulose [63,64], nanocelluloses (e.g., bacterial nanocellulose (BNC) [61,65,66], nanofibrillated cellulose (CNFs) [67,68] and cellulose nanocrystals (CNCs) [69]), chitosan [70,71,72,73], pullulan [71,74,75,76], starch [68], hyaluronic acid [77,78], alginate [79], fucoidan [80,81], agar [82], lysozyme [83,84,85,86], and gelatin [87] (Figure 1) to fabricate films [85,88,89,90,91], membranes [81,92,93,94,95], (nano)composites reference [64,96,97,98,99], coatings [100,101,102,103], nanosystems [24,69,80], patches [78,104,105,106], and microneedles [76,87,107]. These materials can be prepared using different methodologies, from solvent casting [68,89,108] to layer-by-layer technology (LbL) [59,79] for multiple fields of application [5,6,60,61,62,107,109]. The present section provides an overview of our main contributions with respect to natural polymers-based materials derived from cellulose (Table 1), other polysaccharides, and proteins (Table 2).

### 2.1. Cellulose-Based Materials

Cellulose is considered the most abundant biopolymer on the planet, being a constituent of most green plants and algae, and naturally secreted in its pure form by some strains of non-pathogenic bacteria (e.g., *Komagataeibacter*) [66,110]. This polysaccharide is an eminent feedstock for materials development and can be employed in its native state [63,64] as cellulose derivatives, or in the form of nanofibrils (CNFs) reference [85,86,88,89,98,100,101,111], nanorods (CNCs) [69], or three-dimensional hydrogel pellicles (BNC) [77,78,79,81,92,93,94,95,104,105,106,112,113,114,115,116,117,118,119,120,121,122,123,124,125,126,127,128] to manufacture a wide range of materials, as shown in Table 1. Therefore, the vast majority of the works of our research group entails cellulose nanoforms, i.e., cellulose with at least one dimension in the nanoscale, for the development of nanocomposites [98,111,112,113,119,120,124], coatings [100,101], films [85,88,89,123,125], patches [78,79,86,104,105,106,121,122,126,127,128], membranes reference [81,92,93,94,95,114,115,116,117,118], microneedles [77], and nanosystems [69].

Depending on the substrate, the type of material, and the intended function, several manufacturing strategies are accessible for the development of composites and other cellulosic materials. By taking advantage of thermoplastic matrices, e.g., poly(lactic acid) (PLA), poly(hydroxybutyrates) (PHB), and poly(ε-caprolactone) (PCL), biobased composites reinforced with micronized cellulose fibers [64], BNC nanofibers, [112,113] and latex-modified CNFs [98] can be prepared through melt-mixing or hot-pressing approaches. As BNC can be obtained in the form of pellicles with the desired shape, the formulation of functional membranes [81,95] and patches [78,128] is facilitated by the simple diffusion of aqueous solutions of the required (bio)molecules (e.g., drugs [77,104,105,126,127,128], polyelectrolytes [81,95], or other natural polymers [78,81,124]) into its three-dimensional porous network. The introduction of monomers within the BNC structure also allows for in situ free radical polymerization approaches [93,119,120,121,122], either in the presence or absence of a cross-linker, under eco-friendly reaction conditions.

An attractive feature for the obtention of cellulosic-based materials is the effortlessness in the processability of these feedstocks using low-cost and straightforward techniques, namely solvent casting of mixtures of bioactive compounds and nanocelluloses to produce nanocomposites [89], the use of vacuum filtration for the fabrication of nanocomposite films of CNFs with copper nanowires [111] and other polymeric fibers (e.g., lysozyme nanofibrils (LNFs) [85,86]), and the electrostatic assembly of CNFs-based coatings with metallic nanoparticles [100] and metal oxides [101]. In addition to these conventional techniques, some innovative approaches have been addressed by our group. Fonseca et al. [79] prepared chitosan and alginate-coated BNC patches using a spin-assisted LbL methodology. The dexpanthenol-loaded membrane was used as the template for the selective adsorption of the oppositely charged polyelectrolytes, building patches with a different number of layers that influenced the release profile of the drug. From a different angle, Bastante et al. [88] applied supercritical solvent impregnation to produce films of CNFs and bioactive mango leaf extract for active food packaging purposes. This technique makes use of carbon dioxide as the solvent under mild temperature to impregnate active substances in biopolymeric matrices. The biobased films obtained with the CO_2_-assisted technique resulted in improved active properties, namely antioxidant and antimicrobial activities, compared to the properties produced with a standard solvent casting technique.

**Figure 2:**
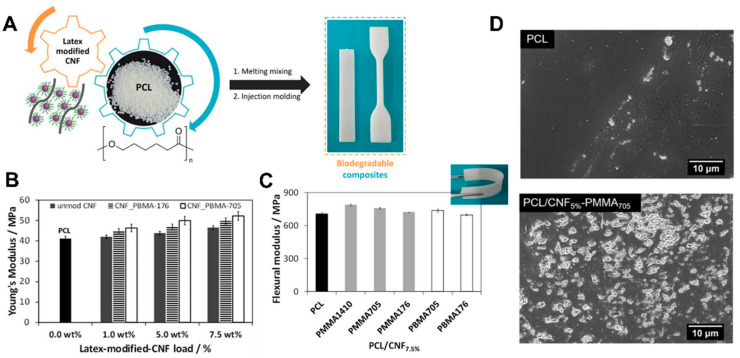
(**A**) Illustration of the preparation of nanocomposites of modified cellulose nanofibers (CNFs) and thermoplastic matrices, in this case poly(ε-caprolactone) (PCL). Plots of Young’s Modulus (**B**) and the flexural modulus (**C**) of PCL and the nanocomposites, showing the improvement of mechanical properties, while retaining flexibility (inset photograph of (**C**). (**D**) Micrographs of the surface of PLC and one of the composites after 10 weeks of enzymatic degradation at 37 °C. Reproduced with permission from [98]. Copyright John Wiley and Sons, 2018.

Owing to their excellent mechanical properties and good thermal stability, cellulose (nano) fibers have been employed as green reinforcing agents for the design of sustainable composites with PLA [64,112], PCL [98,113], and PHB [64] matrices (Figure 2A–C). An interesting issue is that the inclusion of cellulosic fibers, apart from enhancing the mechanical performance of the thermoplastic matrices, may also quicken the degradation rate of the composite materials. As an illustrative example, the deterioration of the surface of latex-modified CNFs/PCL composites [98] under enzymatic conditions was more pronounced when compared to the neat polymer (Figure 2D), highlighting the enhancement of its degradation behaviour.

The exceptional mechanical and thermal properties of bacterial cellulose, allied with its in situ moldability and shape retention, justify the deluge of BNC-based materials (Table 1) for a myriad of applications. The ability to house a variety of molecules in its highly porous nanofibrillar structure can be exploited for the incorporation of drugs and other active compounds [78,104,105,106] for therapeutic and cosmetic purposes. The inclusion of polyelectrolytes [81,93,94,95,114,115,116,117,118] can be used for the formulation of separators for fuel cell applications, and also for the retention of molecules of interest, such as organic dyes [92], giving the structure the ability to be used for environmental remediation. The three-dimensional network of this nanoform is likewise responsible for its selective permeability to gases and liquids [66], a characteristic that is paramount in active packaging applications [123]. Moreover, the high water-binding capacity of BNC (>90% water content) can play a fundamental role in wound management, maintaining a moist environment and absorbing the excess exudate in the wounded site [61].

### 2.2. Other Natural Polymer-Based Materials

Apart from cellulosic substrates, several other polymeric feedstocks of natural origin have been the object of study by our group (Figure 1), in particular polysaccharides derived from marine sources (e.g., chitosan [68,71,72,73,74,90,91,102,103,129], fucoidan [80], and agar [82]) and microorganisms (e.g., pullulan [71,74,75,76,99,108,130,131]), and also some proteins, such as gelatin [87] and lysozyme [108,131] (Table 2). Most of the works deal with the direct shaping of these raw materials into films [71,72,74,75,82,90,91,108,130,131]; however, the design of nanocomposites [68,99,129], coatings [73,102,103], nanosystems [80], and microneedles [76,87] is also described for the above-mentioned natural feedstocks.

Attending to the high filmogenic abilities of polysaccharides such as chitosan and pullulan [20,132], films [71,72,74,75,82,90,91,108,130,131], and composite films [68,129] can be easily obtained via the solvent casting technique. The inclusion of compounds of interest (e.g., metallic nanoparticles [129,130], (nano)fibers [68,99,108,131] and bioactive compounds [75,82,90]) is achieved by direct dispersion or dissolution in the polymer solution before casting. A similar solvent casting process can be used for the manufacture of microneedles using the micromolding technique [76], where the cavities of female molds are progressively filled with the polymer solution, subjected to vacuum or centrifugal forces, and left to dry at room temperature to yield fully filled microneedle arrays [107]. Another strategy for microneedle fabrication involves the use of biopolymer derivatives (e.g., gelatin methacryloyl [87]) that can be cross-linked when exposed to UV irradiation to obtain swellable microneedles for fluid extraction. A different strategy to consider is the microwave-assisted approach used by Pinto et al. [80] for the synthesis of fucoidan/gold nanosystems. With this methodology, monodispersed fucoidan-coated gold nanoparticles (AuNPs) with variable sizes (5.8–13.4 nm) were fabricated after only one minute of exposure to microwave radiation. The partnership of natural polymers with microwave-assisted methodologies results in easier, cheaper, and less-time-consuming protocols for nanoparticle production, compared to traditional synthesis protocols [80,133].

**Figure 3:**
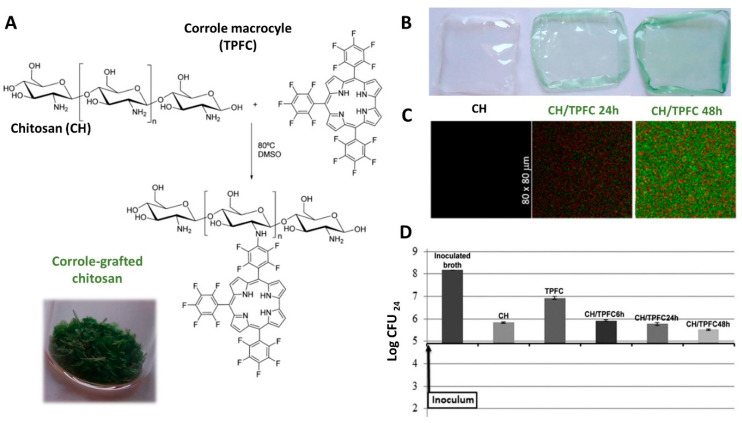
(**A**) Illustration of the grafting of chitosan and 5,10,15-tris(pentafluorophenyl)corrol (TPFC) and inset of a digital photograph of the corrole-grafted chitosan after 48 h reaction. Digital photographs (**B**) and fluorescence lifetime images (**C**) of the neat (CH) and corrole-grafted chitosan films prepared via solvent casting. (**D**) Antibacterial activity of chitosan, TPFC and the corrole grafted-chitosan films against *S. aureus*. Reproduced with permission from [72]. Copyright American Chemical Society, 2016.

Regardless of the type of material produced, the multiplicity of physicochemical and biological properties of the polymeric substrates plays a key role in the properties of the ensuing material. As an example, the combination of LNFs with a pullulan matrix [108] not only positively affected the mechanical properties (Young’s Modulus and elongation at break) of the transparent pullulan films, but was also responsible for the introduction of new functions, specifically antioxidant and antibacterial activities. The use of bioactive molecules and compounds is also a good strategy for the design of materials with these features. For instance, chitosan can be blended with ellagic acid [90], a natural polyphenolic compound, to bestow UV-barrier and antioxidant properties to chitosan films. As another illustrative example, chitosan can be modified through the chemical grafting of corrole macrocycles (e.g., 5,10,15-tris(pentafluorophenyl)corrol, TPFC) [72] to yield transparent films with fluorescent properties (Figure 3A–C), aimed at achieving (bio)imaging and (bio)sensing functions. Moreover, these materials also exhibit a bacteriostatic effect against *Staphylococcus aureus* (Figure 3D).

## 3. Applications of Natural Polymers-Based Materials

Biobased polymeric materials find application in a multitude of fields, as evidenced in Table 1 and Table 2. In the following sections, we briefly discuss the main applications of the materials developed by our research team using cellulose and its nanoforms, other natural polysaccharides, and proteins. Special focus will be given to biopolymeric films designed for active food packaging [75,82,88,89,90,123], ion exchange membranes for fuel cells [81,93,94,95,115,116,117,118], patches for wound healing and drug delivery reference [78,79,86,104,105,106,122,126,127,128], and microneedle systems for drug delivery and fluid uptake [76,77,87], among other applications.

### 3.1. Natural Polymers-Based Films for Active Food Packaging

**Figure 4:**
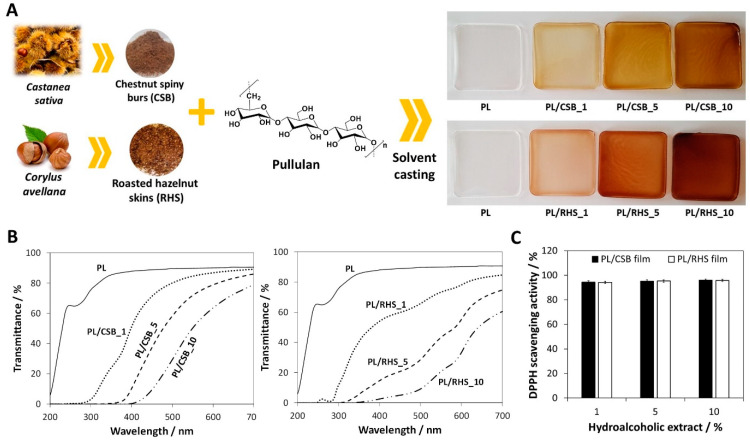
(**A**) Schematic illustration of the incorporation of hydroalcoholic extracts from chestnut spiny burs (CSB) and roasted hazelnut skins (RHS) in a pullulan (PL) matrix via a solvent casting technique and photographs of the prepared films. UV–vis spectra (**B**) and antioxidant activity (**C**) of the PL, PL/CSB, and PL/RHS-based films. Reproduced with permission from [75]. Copyright Elsevier, 2020.

Biopolymeric films for active food packaging are mostly based on nanocelluloses (BNC [123] and CNFs [88,89]) or other film-forming polymeric matrices (e.g., pullulan [74,75,130], chitosan [90] and agar [82]), and are typically loaded with additives with a vast array of functions [62,109], including active components with innate antimicrobial and antioxidant properties, such as silver nanoparticles [130], bioactive ionic liquids [74], lysozyme nanofibrils [108,131], and natural extracts [75,82,88,89,90]. As an illustrative example, Esposito et al. [75] exploited hydroalcoholic extracts from chestnut spiny burs and roasted hazelnut skins, obtained from industrial processes, to prepare homogeneous pullulan films (Figure 4A). The films revealed good mechanical and UV-barrier properties (Figure 4B), allied with high antioxidant activity (ca. 94%, DPPH scavenging activity, Figure 4C), underlining their potential for active food packaging applications. Regarding the topic of active packaging, it is also worth mentioning the recent trends in the design of intelligent packaging options [62,109], which can provide dynamic feedback concerning the condition of packaged food, e.g., monitorization of food humidity levels [123].

### 3.2. Natural Polymers-Based Ion Exchange Membranes for Fuel Cells

**Figure 5:**
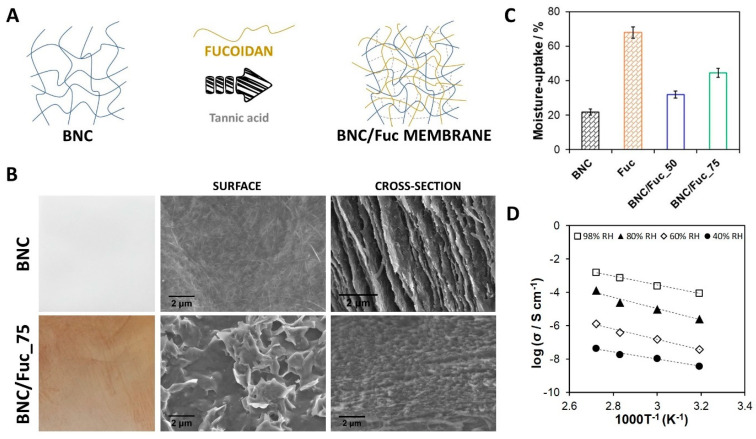
(**A**) Schematic illustration of the preparation of fully biobased bacterial nanocellulose (BNC) and fucoidan (Fuc) membranes with tannic acid as a crosslinker. (**B**) Photographs and micrographs of the nanocellulose surface and cross-section before (BNC) and after (BNC/Fuc_75) inclusion of the sulfated polysaccharide (Fuc) (scale bar: 2 µm). (**C**) Moisture uptake capacity of the membranes. (**D**) Arrhenius-type plot of the through-plane protonic conductivity of the BNC/Fuc_75 membrane. Reproduced with permission from [81]. Copyright Elsevier, 2020.

Nanoscale forms of cellulose have garnered great attention for the design of polymer electrolyte fuel cell (PEFC) components, as recently reviewed by Vilela et al. [60]. In particular, BNC-based materials have been studied for the development of sustainable substitutes of ion-exchange membranes. The lack of intrinsic ionic conductivity of BNC can be surpassed with the introduction of ion-conducting phases in the cellulose nanofibrillar structure [60]. In this sense, BNC has been combined with synthetic polyelectrolytes, such as Nafion^TM^ [116], poly(4-styrene sulfonic acid) [94,114,115], phosphate bearing monomers [93,117], and poly(ionic liquids) [118], to create partially biobased separators. Particularly relevant is the partnership between BNC and natural polyelectrolytes [81,95] for the design of mechanically and thermally robust fully biobased PEMs. Fucoidan and BNC membranes [81] are examples of these materials, prepared via the simple diffusion of aqueous fucoidan solutions into the exopolysaccharide network and subsequent thermal cross-linking with a natural agent, viz. tannic acid (Figure 5A). Micrographs of the surface and cross-section of the membranes disclosed the well-dispersed sulfate moieties of the algal polysaccharide in the BNC network (Figure 5B). The nanocomposite revealed good dynamic mechanical performance (storage modulus ≥ 460 MPa) and thermal-oxidative stability (180–200 °C) in both inert and oxidative atmospheres. Moreover, this fully biobased ion-exchange membrane displayed good moisture-uptake capacity (Figure 5C) and protonic conductivity, with a maximum of 1.6 mS cm^−1^ measured at 94 °C and 98% relative humidity conditions (Figure 5D). In general, fully biobased membranes display lower ionic conductivity (when compared with partially biobased counterparts) associated with a smaller content of moieties that enable ion motion. Nevertheless, the fabrication methodologies are simpler, and the resulting materials are entirely environmentally friendly.

### 3.3. Natural Polymers-Based Patches for Drug Delivery and Wound Healing

**Figure 6:**
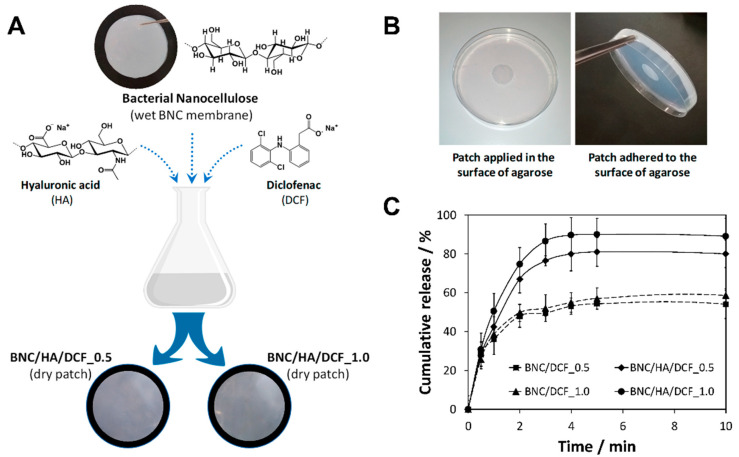
(**A**) Schematic illustration of the preparation of the BNC/HA/DFC patches and photographs of the dried patches. (**B**) Photographs of the adhesion of BNC/HA/DCF patch in an agarose hydrogel skin model. (**C**) Release profile of the drug-loaded patches. Reproduced with permission from [78]. Copyright MDPI, 2020.

Works of BNC [79] and CNFs-based patches [86] have been described as alternatives to non-biodegradable synthetic materials for cutaneous wound healing applications. Aside from the physical protection granted by these systems, the incorporation of antimicrobial agents (e.g., LNFs [86]) or active pharmaceutical ingredients that stimulate cell proliferation (e.g., dexpanthenol [79]) within the patches prevents skin infections and promotes wound closure. In terms of drug-delivery options, nanocellulose patches offer the possibility of self-administering therapeutic molecules using oral, buccal, sublingual, and transdermal routes, which can significantly improve drug bioavailability and minimize side effects by avoiding the first-pass effect on metabolically active tissues of the body [134]. The delivery of pharmacological agents, such as diclofenac [78,104,122,127], ibuprofen [104,105], lidocaine [104,105,126], and other bioactive molecules [79,104,106,128] using nanocellulosic substrates (viz. BNC) continues to be one of the most important areas of study by our research group. These delivery systems can provide a modulatory action of the permeation rates of the different drugs, either for long-term [79] or fast release [78] of active ingredients. For instance, the partnership between BNC, hyaluronic acid, and diclofenac [78] (Figure 6A) resulted in patches that adhered to the moist environment of an oral mucosa skin model (agarose hydrogel) while retaining their mechanical integrity (Figure 6B). In addition to the ability to protect the skin lesions in aphthous stomatitis, the swift local analgesic action of the patches (ca. 90% after 4 min) is highly desirable for the treatment of such acute conditions (Figure 6C).

### 3.4. Natural Polymers-Based Microneedles for Drug Delivery and Fluid Uptake

**Figure 7:**
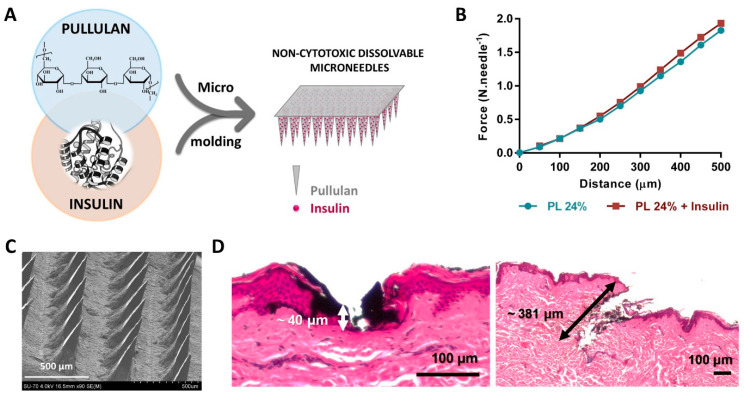
(**A**) Schematic illustration of the production of insulin-loaded pullulan microneedles. (**B**) Force–displacement curves of neat (PL 24%) and drug-loaded (PL 24% + insulin) microneedles. (**C**) SEM micrograph (scale bar: 500 μm) and (**D**) histological cross-sections of human abdominal skin after insertion of the pullulan microneedle patch with insulin. Reproduced with permission from [76]. Copyright Elsevier, 2020.

Microneedles (MNs) have risen as an efficient, minimally invasive, and painless method for transdermal delivery of an assortment of drugs and bioactive compounds [107]. In this field, we report the fabrication of pullulan MNs for the transdermal delivery of insulin [76] (Figure 7A) and hyaluronic acid MNs with a BNC backing layer for the delivery of rutin [77], using a micromolding technique. In both cases, the casting of the polymeric solutions resulted in needles with good mechanical performance (Figure 7B), able to meet the force threshold required for skin insertion (≈0.15 N.needle^−1^). Moreover, penetration studies using both a skin model and human skin (in vitro) proved the ability of the microneedles to successfully perforate the outmost layer of the skin, i.e., the *stratum corneum*, and create pathways across the skin without reaching the nerves (Figure 7C,D).

Microneedle patches can also be designed for interstitial skin fluid uptake, which is rich in biomarkers of clinical relevance [107]. Fonseca et al. [87] took advantage of the swelling ability of a gelatin derivative to produce MNs capable of extracting urea, a metabolite of great importance for the management of kidney diseases. When exposed to ex vivo human abdominal skin, a single patch was able to extract 3.0 ± 0.7 mg of fluid. Overall, microneedle systems are associated with quicker diagnosis, the onset of therapeutics, and less pain and discomfort, which can be of significance for low-compliant patients such as the elderly [107].

### 3.5. Natural Polymers-Based Materials for Other Applications

In addition to the examples listed above, our group has also been exploiting natural polymers for several other emerging fields of application, ranging from electronics to environmental remediation. For example, the formulation of biopolymeric chitosan coatings loaded with corrosion inhibitors [73,102,103] was adopted for the protection of metallic substrates in corrosive environments. In a different approach, flexible electroconductive substrates with potential application in electronic devices, energy storage, or sensors were devised through the inclusion of copper nanowires [111] or organic conductive polymer solutions (e.g., poly(3,4-ethylenedioxythiophene): polystyrene sulfonate (PEDOT:PSS) [125]) in nanocellulosic substrates. The functionalization of biopolymers can also be exploited to impart luminescence or (bio)imaging functions to the ensuing materials; for instance, the blend of chitosan and pullulan films with lanthanopolyoxometalates [71], the chemical grafting of corrole macrocycles in a chitosan matrix [72], and the adsorption of a chitosan-derivative containing fluorescein isothiocyanate [69] on the surface of CNCs. In the field of water remediation, works related to the manufacture of bio-sorbent membranes of BNC/poly(2-methacryloyloxyethyl phosphorylcholine) [92] and CNFs/LNFs [85] have been suggested for the removal of organic dyes (methylene blue, methylene orange) and mercury from contaminated waters, respectively.

## 4. Perspectives and Conclusions

As illustrated in this review, nature offers a high variety of polymeric feedstocks with inherent chemical and biological properties that can be used to fabricate novel functional materials with enhanced and customizable properties, potentiating their application in several areas. Biobased nanofibrils obtained from polysaccharides, such as cellulose and proteins (e.g., lysozyme), display excellent mechanical strength and are commonly employed as reinforcement agents to improve the mechanical properties of materials [98,108,113]. Polymers with good film-forming abilities, such as chitosan and pullulan, can be casted into different shaped materials such as films, membranes, and microneedle patches [75,76,87,90]. Moreover, many reports demonstrate the use of BNC as a nanostructured membrane (Table 1), due to ease of production in the final desired shape and the high purity associated with this nanoform, which is of relevance for some applications such as wound healing and drug delivery.

The judicious selection of functional molecules and compounds to incorporate in the biopolymeric matrices can yield partially or fully biobased materials with distinct properties and, therefore, different applications. For instance, the combination of pullulan with silver nanoparticles translates into films with good antimicrobial action that could be used for food packaging systems [130]; its blend with insulin provides a new platform for the transdermal delivery of the peptide hormone in diabetic patients [76]; and the introduction of lanthanopolyoxometalates results in luminescent materials [71]. Some natural polymers can serve a dual purpose in the final material. Fucoidan can be used as a green agent to reduce gold solutions into zero-valent gold nanoparticles and simultaneously endow anti-cancer properties that are intrinsically related to the biological activities of the sulfated polysaccharide [80]. Similarly, lysozyme nanofibrils can provide mechanical reinforcement of polymeric matrices and impart antimicrobial properties that are desirable in active packaging and biomedical applications [108]. As a result, the proper design of functional biobased polymeric materials increases their application in high-tech fields, including active and intelligent food packaging [75,89,123], smart coatings [73,101], drug delivery [76,78,105], imaging [69], water remediation [85,92], sensors [111,125], and fuel cells [81,95,115].

These reports highlight the great potential of natural polymers in the development of biobased materials that could, eventually, replace the fully synthetic materials to which we are accustomed. Taking into consideration the concept of the triple-bottom-line approach, the development of such ecological materials should also be closely related to social and economic balance. Clearly, the partial or even full transition to more biobased platforms is necessary to guarantee that the sources of these polymers do not compete with the food supply chain and are cost-effective, particularly with respect to exopolysaccharides such as BNC and pullulan, which are particularly expensive when compared to other natural polymers. In this sense, the use of agroforest waste products for the extraction of biopolymers [135] or the supplementation of the culture medium of microorganisms [136] is a viable alternative for the sustainable obtention of some of these natural feedstocks and could encourage their utilization on a larger-scale. Moreover, it can aid in the valorisation of wastes, boosting the profit of related industries and narrowing the production loop towards a zero-waste economy [137].

As Noyori stated in his Nobel Prize acceptance speech, “Green chemistry is not a mere catchphrase. It is an indispensable principle of chemical research that will sustain our civilized society in the twenty-first century and further into the future” [138]. Indeed, in this appraisal, we delved into a few examples related to a particular principle from the dozen enumerated by Paul Anastas, and we hope that the great prospects natural polymers have to offer for the development of functional and innovative materials in a variety of fields are clear. Nonetheless, the indissociable nature of the stated principles is evident. As most of the materials can be obtained using mild solvents (usually water) and simple fabrication techniques (e.g., electrostatic assembly [100,101], solvent-casting [75,89], and micromolding [76,77]), the use of natural polymers is intrinsically related to Principles 5 (“Safer solvents and auxiliaries”) and 10 (“Design for degradation”). In other works, we explored the substitution of chemical reducing agents with biological extracts [80], which is also in line with Principle 5, and the use of advanced manufacturing techniques and processes (e.g., supercritical solvent impregnation [88] and microwave-assisted synthesis [80]) that reduce the need for hazardous solvents (Principle 5) and decrease energy consumption (Principle 6). The need for environmentally friendly approaches for material development is inevitable and, as such, all principles of Green Chemistry (although not new) should be taken into consideration when planning the experimental design for laying out sustainable practices for generations to come.

## Figures and Tables

**Table 1:** Examples of cellulose and nanocellulose-based materials developed by the BioPol4fun research group.

Cellulosic Substrate	Other Components	Type of Material	General Features	Application	Refs.
BNC	PLA	Nanocomposites	Methodology: Melt-mixing Mechanical properties: Improvement of YM up to 40% Thermal stability: Increase up to 14 °C, in the maximum degradation temperature	–	[112]
BNC	PCL	Nanocomposites	Methodology: Supplementation of the BNC culture medium with PCL powder and hot-pressing Thermal stability: Up to 200 °C Other characteristics: Decrease of the storage modulus with increasing amounts of PCL highlights the reinforcement role of BNC in the nanocomposites	–	[113]
BNC	PGMA	Nanocomposites	Methodology: in situ free radical polymerization Thermal stability: Up to 270 °C Water uptake capacity: Up to 32.8%, after 48 h Storage modulus: min. 80 MPa at 200 °C Other characteristics: Post-modification via acid-catalyzed hydrolysis improved the water-uptake capacity (up to 222%, after 48 h)	–	[119,120]
BNC	Pullulan	Nanocomposites	Methodology: Solvent casting Mechanical properties: Improvement of YM up to 8000% Thermal stability: Up to 40 °C, in the maximum degradation temperature	–	[124]
BNC	PSBMA	Films	Methodology: One-pot polymerization Water uptake capacity: Up to 559%, after 48 h Antibacterial activity: Staphylococcus aureus (4.3-log CFU reduction) and Escherichia coli (1.1-log CFU reduction) Ionic conductivity: Max. 1.5 mS cm^−1^, at 94 °C and 98% RH Other characteristics: UV-light barrier properties	Active and intelligent food packaging	[123]
BNC	PEDOT:PSS	Films	Methodology: Ink-jet printing of electrodes in BNC Other characteristics: BNC substrate lowers the PEDOT:PSS impedance and minimizes the effects of the 1/f^2^ noise; Ability to record low-frequency signals from non-electrogenic cells (glioma cells)	Sensors	[125]
BNC	PSSA	Membranes	Methodology: in situ free radical polymerization Ionic conductivity: max. 185 mS cm^−1^, at 94 °C and 98% RH Fuel cell tests: max. of ca. 40 mW cm^−2^ at 125 mA cm^−2^ Microbial fuel cell performance (*Shewanella frigidimarina*): max. power density of 2.42 mW m^−2^, open-circuit voltage of 0.436 V, internal resistance of 15.1 kΩ	Ion exchange membranes for fuel cells	[94,114,115]
BNC	Nafion^TM^	Membranes	Methodology: Diffusion of Nafion^TM^ in the BNC matrix Ionic conductivity: 140 mS cm^−1^, at 94 °C and 98% RH	Ion exchange membrane for fuel cells	[116]
BNC	PMOEP	Membranes	Methodology: in situ free radical polymerization Water uptake capacity: Up to 206%, after 48 h Ionic conductivity: max. 100 mS cm^−1^, at 80 °C and 98% RH	Ion exchange membrane for fuel cells	[117]
BNC	PMACC	Membranes	Methodology: in situ free radical polymerization Water uptake capacity: Up to 2057%, after 48 h Ionic conductivity: max. 10 mS cm^−1^, at 94 °C and 98% RH	Ion exchange membrane for fuel cells	[118]
BNC	P(bis-MEP)	Membranes	Methodology: in situ free radical polymerization Water uptake capacity: Up to 155%, after 48 h Ionic conductivity: max. 30 mS cm^−1^, at 80 °C and 98% RH	Ion exchange membrane for fuel cells	[93]
BNC	Fucoidan	Membranes	Methodology: Diffusion of aqueous solutions in the BNC matrix Moisture uptake capacity: ca. 45%, at 98% RH after 48 h Ionic conductivity: max. 1.6 mS cm^−1^, at 94 °C and 98% RH	Ion exchange membrane for fuel cells	[81]
BNC	Lignosulfonates	Membranes	Methodology: Diffusion of aqueous solutions in the BNC matrix Moisture uptake capacity: ca. 78%, at 98% RH after 48 h Ionic conductivity: max. 23 mS cm^−1^, at 94 °C and 98% RH	Ion exchange membrane for fuel cells	[95]
BNC	PMPC	Membranes	Methodology: in situ free radical polymerization Water uptake capacity: 627–912%, for 48 h at different pH values (2.1, 7.4, 12) Antibacterial activity: *S. aureus* (4.3-log CFU reduction) and *E. coli* (1.8-log CFU reduction) Ionic dye adsorption: Up to 4.5 mg g^−1^	Water remediation (removal of organic dyes)	[92]
BNC	Hyaluronic acid	Microneedles	Methodology: Micromolding and BNC backing layer Antioxidant activity: IC_50_ of 23.24 μg·mL^−1^ (DPPH scavenging activity) Cumulative release: ca. 94% rutin after 6.5 h (ex vivo skin) Percutaneous permeation (in vitro): 9.85%, after 12 h Other characteristics: Penetration up to 99.3 µm depth (ex vivo skin insertion); Non-cytotoxic towards HaCaT cells	Drug delivery (rutin)	[77]
BNC	PMETAC	Patches	Methodology: in situ free radical polymerization Water uptake capacity: Up to 873%, in distilled water after 48 h Antifungal activity: Candida albicans (4.4-log CFU reduction) Other characteristics: UV-light barrier properties; Non-cytotoxic towards HaCaT cells	Treatment of fungal infections	[121]
BNC	PMGly	Patches	Methodology: in situ free radical polymerization Water uptake capacity: Up to 23.5% (pH 2.1) and 77.8% (pH 7.4) after 24 h Cumulative release: pH dependent – ca. 9%, after 20 h at pH 2.1 and ca. 70%, after 1 h at pH 7.4 Other characteristics: Non-cytotoxic towards HaCaT cells	Drug delivery (diclofenac)	[122]
BNC		Patches	Methodology: Diffusion of aqueous solutions in the BNC matrix Moisture uptake capacity: Up to 26.0% (BNC/caffeine), 36.3% (BNC/lidocaine), 12.3% (BNC/ibuprofen) and 31.3% (BNC/diclofenac), at 75% RH, 40 °C for 3 months Cumulative release: 100% after 5 min (caffeine and lidocaine patches), 30 min (ibuprofen patch) and 15 min (diclofenac patch), in PBS Other characteristics: No significant changes in the cumulative release were observed after the accelerated stability tests; Good in vivo compatibility	Drug delivery (caffeine, lidocaine, ibuprofen, diclofenac)	[104]
BNC		Patches	Methodology: Diffusion of aqueous solutions in the BNC matrix Percutaneous permeation (in vitro): 31.4 μg cm^−2^ h^−1^ (lidocaine) and 11.9 μg cm^−2^ h^−1^ (ibuprofen)	Drug delivery (lidocaine, ibuprofen)	[105]
BNC		Patches	Methodology: Diffusion of aqueous solutions in the BNC matrix Cumulative release: >90% after 20 min, in PBS Percutaneous permeation (in vitro): 31.35 μg cm^−2^ h^−1^	Drug delivery (lidocaine)	[126]
BNC		Patches	Methodology: Diffusion of aqueous solutions in the BNC matrix Water uptake capacity: Up to 1400%, in PBS after 8 h Cumulative release: ca. 90% after 10 min, in PBS Percutaneous permeation (in vitro): 1.21 μg cm^−2^ h^−1^	Drug delivery (diclofenac)	[127]
BNC	Alginate + Chitosan	Patches	Methodology: LbL assembly Moisture uptake capacity: 240–250%, at 100% RH, 25 °C for 22 h Cumulative release: ca. 95% after 16 h (patch with 5 layers) up to ca. 65% after 90 h (patch with 21 layers) Antibacterial activity: *S. aureus* (3.2-log CFU reduction) Other characteristics: Non-cytotoxic towards HaCaT cells; Good cell migration capacity on wound healing assay	Wound healing Drug delivery (dexpanthenol)	[79]
BNC	Caffeine	Patches	Methodology: Diffusion of aqueous solutions in the BNC matrix Water uptake capacity: Up to 284%, in PBS solution, after 2 h Cumulative release: max. 80% after 15 min, in PBS solution Percutaneous permeation (in vitro): 2.55 μg cm^−2^ h^−1^	Dermal care	[128]
BNC	ILs + vitamin B	Patches	Methodology: Diffusion of aqueous solutions in the BNC matrix Water uptake capacity: Up to 1697%, in PBS solution, after 24 h Cumulative release: At least 66% after 5 min, in PBS solution Other characteristics: Non-cytotoxic towards HaCaT cells	Dermal care	[106]
BNC	Hyaluronic acid	Patches	Methodology: Diffusion of aqueous solutions in the BNC matrix Water uptake capacity: Up to 484%, in agarose skin model Cumulative release: max. 90% after 4 min, in simulated salivary fluid Other characteristics: Non-cytotoxic towards HaCaT cells	Drug delivery (diclofenac)	[78]
Cellulose fibres	CaCO_3_	Composites	Methodology: in situ synthesis Mechanical properties: Incorporation of the CaCO_3_/cellulose fibres improved the stiffness of polyethylene films	–	[63]
Cellulose fibres	PLA and PHB	Composites	Methodology: Melt-mixing Mechanical properties: Increase in YM up to 5.83 GPa, elongation at break < 3.5%, tensile strength up to 72.9 MPa and flexural modulus up to 8.1 GPa Thermal stability: Increase up to 44 °C in the maximum thermal degradation temperature Other characteristics: Decrease in water uptake, compared to neat matrices and PLA or PHB with non-micronized fibres	–	[64]
CNCs	Chitosan derivative with folic acid and fluorescein isothiocyanate	Nanosystems	Methodology: Physical adsorption Other characteristics: Fluorescent nanosystems; Non-cytotoxic towards MDA-MB-231 breast cancer cells; Anti-proliferative effect suggested by exometabolomics analysis	–	[69]
CNFs	AgNPs	Coatings	Methodology: Electrostatic assembly Air permeability: Up to 9.54 nm Pa^−1^ s^−1^ Antibacterial activity: *S. aureus* and *Klebsiella pneumoniae* (total inhibition after 24 h)	–	[100]
CNFs	ZnO	Coatings	Methodology: Electrostatic assembly Air permeability: Up to 10.81 nm Pa^−1^ s^−1^ Antibacterial activity: *S. aureus* (up to 3.4-log CFU reduction), *Bacillus cereus* (up to 3.5-log CFU reduction) and K. pneumoniae (total inhibition after 24 h, at [ZnO] >2%)	–	[101]
CNFs	PCL	Nanocomposites	Methodology: Melt-mixing Thermal stability: 335–340 °C Mechanical properties: YM of 43.6–52.3 MPa Other characteristics: Degradable under enzymatic conditions	–	[98]
CNFs	CuNWs	Nanocomposites	Methodology: Vacuum filtration Mechanical properties: YM of 2.62–4.72 GPa Electrical conductivity: Up to 5.43 × 10^4^ S m^−1^	–	[111]
CNFs	LNFs	Films	Methodology: Vacuum filtration Hg(II) removal: ca. 99% after 24 h of contact time, at pH 11	Water remediation (mercury removal)	[85]
CNFs	Arabinoxylans + ferulic acid or feruloylated arabinoxylo-oligosaccharides	Films	Methodology: Solvent casting Antioxidant activity: Up to 90% (DPPH scavenging activity) Antibacterial activity: *S. aureus* (>3-log CFU reduction) and *E. coli* (up to 3-log CFU reduction) Antifungal activity: *C. albicans* (1.1-log CFU reduction) Other characteristics: UV-light barrier properties	Active food packaging	[89]
CNFs	Mango leaf extract	Films	Methodology: Supercritical solvent impregnation Antioxidant activity: ca. 84% (DPPH scavenging activity) Antibacterial activity: *S. aureus* (max. growth inhibition ≈ 37%) and *E. coli* (max. growth inhibition ≈ 91%) Other characteristics: UV-light barrier properties	Active food packaging	[88]
CNFs	LNFs	Patches	Methodology: Vacuum filtration Antioxidant activity: 76–79% (DPPH scavenging activity) Antibacterial activity: *S. aureus* (3.5-log CFU reduction) Other characteristics: Non-cytotoxic towards L929 fibroblast cells; Good cell migration capacity on wound healing assay	Wound healing	[86]

Abbreviations: AgNPs: silver nanoparticles; BNC: bacterial nanocellulose; CFU: colony-forming unit; CNCs: cellulose nanocrystals; CNFs: nanofibrillated cellulose; CuNWs: copper nanowires; DPPH: 2,2-diphenyl-1-picrylhydrazyl; ILs: ionic liquids; LbL: layer-by-layer technology; LNFs: lysozyme nanofibrils; P(bis-MEP): poly(bis[2-(methacryloyloxy)ethyl] phosphate); PBS: phosphate-buffered saline; PCL: poly(ε-caprolactone); PEDOT:PSS: poly(3,4-ethylenedioxythiophene):polystyrene sulfonate; PGMA: poly(glycidyl methacrylate); PHB: poly(hydroxybutyrate); PLA: poly(lactic) acid; PMACC: poly(methacroylcholine chloride); PMETAC: poly([2-(methacryloyloxy)ethyl]trimethylammonium chloride); PMGly: poly(*N*-methacryloyl glycine); PMOEP: poly(methacryloyloxyethyl phosphate); PMPC: poly(2-methacryloyloxyethyl phosphorylcholine); PSBMA: poly(sulfobetaine methacrylate); PSSA: poly(4-styrene sulfonic acid); RH: relative humidity; UV: ultraviolet; YM: Young’s Modulus.

**Table 2:** Examples of other polysaccharide- and protein-based materials developed by the BioPol4fun research group.

Natural Polymer	Other Components	Type of Material	General Features	Application	Refs.
Agar	*Opuntia ficus-indica* cladodes powder	Films	Methodology: Solvent casting WVTR: 12.67–14.17 g h^−1^ m^−2^ Other characteristics:UV-light barrier properties	Food packaging	[82]
Chitosan	MBT	Coatings	Methodology: Functionalization of the chitosan matrices Other characteristics: Corrosion protection towards Al alloy 2024; pH-dependent release	Anti-corrosion	[73,102]
Chitosan	Cerium (III) nitrate	Coatings	Methodology: Solvent casting Other characteristics: Corrosion protection towards Al alloy 2024; Self-healing ability	Anti-corrosion	[103]
Chitosan	Starch, BNC/CNFs fibres	Nanocomposites	Methodology: Solvent casting Thermal stability: Increase up to 15 °C, in the maximum degradation temperature Mechanical properties: YM up to 20 MPa Antimicrobial activity: *S. aureus* (up to 3-log CFU reduction)	–	[68]
Chitosan	AgNPs	Nanocomposites	Methodology: Solvent casting Antibacterial activity: *S. aureus* (up to 3-log CFU reduction), *Klebsiella pneumoniae* (up to 5.5-log CFU reduction) and *E. coli* (up to 4.5-log CFU reduction)	–	[129]
Chitosan	Ellagic acid	Films	Methodology: Solvent casting WVP: 2.82–3.70 g mm m^−2^ day^−1^ kPa^−1^ Antioxidant activity: *ca.* 28% (DPPH scavenging activity) Antibacterial activity: *S. aureus* and *Pseudomonas aeruginosa* (total inhibition after 24 h) Other characteristics:UV-light barrier properties	Active food packaging	[90]
Chitosan	Meso-tetraarylporphyrins	Films	Methodology: Solvent casting Antibacterial activity: Inhibition of *Listeria innocua* attachment (up to 6-log CFU reduction) and biofilm formation Other characteristics: Films are photostable and able to generate singlet oxygen under visible light irradiation	Photodynamic antifouling materials	[91]
Chitosan	Corrole macrocycle	Films	Methodology: Chemical grafting and solvent casting Antibacterial activity: *S. aureus* (2-log CFU reduction) Other characteristics: Films exhibit fluorescence	–	[72]
Chitosan	LnPOMs	Films	Methodology: Solvent casting Other characteristics: Films exhibit fluorescence under UV irradiation	–	[71]
Chitosan	Cholinium carboxylate ILs	Films	Methodology: Solvent casting Antibacterial activity: *S. aureus* (6.5-log CFU reduction) and *K. pneumoniae* (6.4-log CFU reduction) Other characteristics: Plasticizing effect of ILs	–	[74]
Fucoidan	AuNPs	Nanosystems	Methodology: Microwave-assisted synthesis Antitumoral activity: MNT-1, HepG2 and MG-63 tumour cell lines (reduction of cell viability up to 90%, at 72 h) Other characteristics: Cellular uptake confirmed using flow cytometry/dark-field imaging	–	[80]
Gelatin		Microneedles	Methodology: Photo-cross-linking and micromolding Fluid uptake: ca. 3.7 mg fluid/patch Urea recovery: >98%, in agarose skin model Other characteristics: Penetration up to 237 µm depth (*ex vivo* skin insertion); Non-cytotoxic towards HaCaT cells	ISF extraction for urea monitoring	[87]
Pullulan	CNFs	Nanocomposites	Methodology: Solvent casting Mechanical properties: Improvement of YM up to 5500% Thermal stability: Increase up to 20 °C, in the maximum degradation temperature	–	[99]
Pullulan	LnPOMs	Films	Methodology: Solvent casting Other characteristics: Films exhibit fluorescence under UV irradiation	–	[71]
Pullulan	Cholinium carboxylate ILs	Films	Methodology: Solvent casting Antibacterial activity: *S. aureus* (2.6-log CFU reduction) and *K. pneumoniae* (3.5-log CFU reduction), only for the films with cholinium citrate Other characteristics: Plasticizing effect of ILs	–	[74]
Pullulan	AgNPs	Films	Methodology: Solvent casting Antifungal activity: *Aspergillus niger* (76% inhibition)	–	[130]
Pullulan	LNFs	Films	*Methodology***:** Solvent casting Antioxidant activity: *ca.* 77% (DPPH scavenging activity) Antibacterial activity: *S. aureus* (3.2-log CFU reduction)	Active food packaging	[108,131]
Pullulan	Extracts from chestnut spiny burs and roasted hazelnut skins	Films	Methodology: Solvent casting Antioxidant activity: *ca.* 94% (DPPH scavenging activity) Antibacterial activity: *S. aureus* (4-log CFU reduction) Other characteristics: UV-light barrier properties	Active food packaging	[75]
Pullulan		Microneedles	Methodology: Micromolding Cumulative release: *ca.* 87% insulin after 2 h (*ex vivo* skin) Other characteristics: Penetration up to 381 µm depth (*ex vivo* skin); Non-cytotoxic towards HaCaT cells	Drug delivery (insulin)	[76]

Abbreviations: AgNPs: silver nanoparticles; AuNPs: gold nanoparticles; BNC: bacterial nanocellulose; CFU: colony-forming unit; CNFs: nanofibrillated cellulose; DPPH: 2,2-diphenyl-1-picrylhydrazyl; ILs: ionic liquids; ISF: interstitial skin fluid; LNFs: lysozyme nanofibrils; LnPOMs: lanthanopolyoxometalates; MBT: 2-mercaptobenzothiazole; UV: ultraviolet; WVP: water vapour permeability; WVTR: water vapour transmission rate; YM: Young’s Modulus.
